# Author’s response: Viral load decline time offers theoretical insight rather than practical value

**DOI:** 10.2807/1560-7917.ES.2025.30.9.2500155

**Published:** 2025-03-06

**Authors:** Jakob Jonnerby, Ajit Lalvani, on behalf of all co-authors of the original article

**Affiliations:** 1 NIHR Health Protection Research Unit in Respiratory Infections, National Heart and Lung Institute, Imperial College London, London, United Kingdom; 2 https://www.eurosurveillance.org/content/10.2807/1560-7917.ES.2025.30.6.2400234

**Keywords:** SARS-CoV-2, influenza A, viral kinetics, infectiousness, transmission risk, viral decline time

**To the editor:** By analysing two prospective, longitudinal cohort studies of severe acute respiratory syndrome coronavirus 2 (SARS-CoV-2) and influenza A virus index cases and their respective household contacts, we identified viral load (VL) decline time (the time for the VL to reduce by a factor of e of c.a. 2.72) as a potential marker for infectiousness and transmission risk [1]. The recent letter to the editor raises some interesting questions, which we further address below.

First, the authors of the letter correctly state that several factors determine VL kinetics of respiratory viruses, including genetic diversity and variations in immune response, in part due to heterogeneity of previous exposure. We do not exclude that such factors likely influence VL kinetics. However, this does not negate the hypothesis we investigated, namely that aspects of VL kinetics, such as VL decline time, could correlate with infectiousness at a population level, even though they obviously would not be expected to explain all inter-individual variation in infectiousness. Indeed, the correlation we discovered is also mechanistically plausible as we observed a strong association between VL decline time and the total amount of shedding of infectious virus, quantified using the number of plaque forming units (PFU). As we state in the Introduction, PFUs are an important determinant of infection risk in challenge models [1-3]. Furthermore, as we highlight in the Discussion, the index case VL has previously been shown to correlate with transmission risk in several independent studies of SARS-CoV-2 [4-7] and influenza A virus [8]. Our results are therefore complementary to these previous studies in showing that VL kinetics are important for transmission risk, while additionally demonstrating that the VL decline time may be a more sensitive marker of infectiousness than the VL at a single time point as it is less dependent on the timing of sample collection. This is shown in Figures 1 and 4 of the article [1].

Second, the importance of accounting for SARS-CoV-2 variants and influenza virus subtypes is carefully considered in the Discussion as these influence both the VL kinetics and the transmission risk. The detailed epidemiological and virological data collected in both cohort studies described in our report allowed us to ascertain both the SARS-CoV-2 variant and influenza A virus subtype in most cases. By adjusting for either variant or subtype, we were able to mitigate this potential source of bias in our analyses [1].

Third, the limitation of using VL decline time to predict transmissions on an individual level was acknowledged in the Discussion: “*The applicability of RNA VL decline time to predict, and enable prevention of, transmission on an individual level is limited as a proportion of transmissions will occur early during infection, before RNA VL decline time can be measured*”. We also suggested potential population-level applications of the VL decline time as a marker of infectiousness in public health practice: “*the VL decline time could help identify immune markers associated with infectiousness, potentially informing new medical interventions against transmission*” and *“as a tool to improve understanding of infection dynamics at a population level*” [1].

Finally, the small sample size and attendant statistical uncertainty of our study were candidly addressed in the Discussion: “*However, the 95% CrIs for the relative increase in SAR with longer VL decline times were found to be wide and include zero, indicating uncertainty in the estimated parameter values*” and “*Larger independent studies would help to confirm our findings*”. We also recognised the potential for misclassification of transmission pairs: “*We cannot exclude the possibility that some contacts were the source of infection, or became infected from outside the household, but this likely constitutes a minority of cases as epidemiological linkage confirmed the index-contact pairings*” [1]. 
